# The Effect of a Local Injection of Flurbiprofen Ester Microspheres on Systemic Inflammatory Model Rats With a Closed Femoral Shaft Fracture

**DOI:** 10.3389/fphar.2022.769577

**Published:** 2022-03-24

**Authors:** Hui-Ming Peng, Ke Xiao, Wei Zhu, Ying-Jie Wang, Yan-Yan Bian, Wei Wang, Wen-Wei Qian, Xi-Sheng Weng

**Affiliations:** ^1^ Department of Orthopedics, Peking Union Medical College Hospital, Chinese Academy of Medical Sciences and Peking Union Medical College, Beijing, China; ^2^ Department of Orthopedics, West China Hospital, Sichuan University, Chengdu, China

**Keywords:** nonsteroidal anti-inflammatory analgesics, flurbiprofen ester microspheres, systemic inflammatory, femoral shaft fracture, multimodal analgesia

## Abstract

**Background:** Periarticular injections with a combination of local anesthetics, non-steroidal anti-inflammatory analgesics (NSAIDs), and epinephrine are becoming increasingly popular in the perioperative analgesia of artificial joint replacement. However, data on the efficacy and safety of local injection NSAIDs are still scarce. The purpose of this study was to investigate the efficacy and safety of a local injection of Flurbiprofen Ester Lipid microspheres into the inflammatory model of femoral shaft closed fractures in rats.

**Methods:** A systemic inflammatory model was induced in SD rats (60) by closed femoral shaft fracture; 12 non-fractured rats were used as the blank control group (group A). The systemic inflammation model of 60 rats was divided into 5 groups (12 in each group); Group B: intramuscular injectionof the same amount of normal saline at different time points as a negative control; Group C: intravenous injection of Flurbiprofen Ester microspheres (4.5 mg/kg) at different time points; Group D: intramuscular injection of Flurbiprofen Ester microspheres (2.25 mg/kg) at different time points; Group E: intramuscular injection of Flurbiprofen Ester microspheres (4.5 mg/kg) at different time points; Group F: intramuscular injection of Flurbiprofen Ester microspheres (9 mg/kg) at different time points. The behavioral test observed the behavior of the rats. Then, the inflammation factors of CRP, IL-6, COX-1, COX-2 and TNF-αby ELISA were recorded.

**Results:** Through the behavioral test it could be found that the effect of the intramuscular and intravenous injections of Flurbiprofen Ester microspheres was similar. Fracture rats with a local injection of Flurbiprofen Ester microspheres showed lower inflammation levels measured by COX-1, CRP, and TNF-α compared with the control group. Pathological sections at 24, 48, and 96 h after surgery did not display any local muscle necrosis at the local injection site. These findings suggested that a Flurbiprofen Ester microsphere muscular injection exhibited a similar effect to an intravenous injection.

**Conclusion:** The local injection of Flurbiprofen Ester microspheres significantly reduced the inflammatory response in fracture rats and did not increase the risk of muscle necrosis, suggesting its feasibility in local injection analgesia.

## Introduction

Multimodal analgesia has become the standard analgesia regimen for the postoperative treatment of total knee and total hip arthroplasty (Elmallah et al., 2018; Yu et al., 2018). Excellent perioperative analgesia helps to accelerate postoperative rehabilitation, improve patient satisfaction, and obtain satisfactory joint function (Lavernia et al., 2008). A periarticular injection is a critical component of the multimodal pain management program (Zhang et al., 2018). The current clinical use of multiple mixed analgesic injections aims to improve the analgesic effect, extend the use time, and reduce the local inflammatory response (Ross et al., 2017). However, there is still no agreement on the composition of the mixed drug regimen for injection around the hip and knee joint, so there is currently little standardization of the mixed drug regimen in clinical practice.

Non-steroidal anti-inflammatory drugs (NSAIDs) inhibit cyclooxygenase (COX), and prevent the body from producing proinflammatory mediators, such as prostaglandins (Lee et al., 1999). Peripheral prostaglandins sensitize pain receptors, increase the firing rate of nerve fibers, and increase the duration of action potentials, leading to increased hyperalgesia in the brain (Romsing and Moiniche, 2004). Wen et al. (2013) reported that the peripheral administration of NSAIDs alleviated both mechanical joint pain and hyperalgesia, and reduced the sensitivity of pain receptors in animal models of arthritis. In theory, a periarticular injection of a mixed drug containing NSAIDs may help reduce surgical inflammation and, ultimately, postoperative pain. However, after the peripheral administration of NSAIDs, the following questions remain: 1) whether it can reduce the level of systemic inflammatory response; 2) whether a local injection can increase the risk of muscle necrosis at the injection site. 3) is there a theoretical risk of increased levels of NSAIDs in plasma? Although it has been reported (Pinsornsak et al., 2017) that the addition of ketorolac to the mix for an injection around the knee decreased pain scores and opioid consumption, at present all commercially available NSAIDs drugs do not include a topical injection as a method of use.

Flurbiprofen Ester lipid microspheres (Kaifen, Beijing taide pharmaceutical co., ltd.) is a non-steroidal targeted analgesic drug. Flurbiprofen axeti is a prodrug of flurbiprofen, which is obtained by esterification of flurbiprofen. Flurbiprofen is a widely used non steroidal anti-inflammatory drug. It plays an anti-inflammatory and analgesic role by inhibiting the activity of prostaglandin synthase cyclooxygenase. This product has powerful anti-inflammatory, analgesic and antipyretic functions, small therapeutic dose and low side effects. Ester microspheres is a microsomal dispersion system with fatty oil as soft matrix and encapsulated by phospholipid membrane. The average diameter is 200 nm. The outer membrane is lecithin and the inner layer is soft matrix oil, in which lipid soluble drugs are encapsulated. It can reduce the synthesis of prostaglandin through the peripheral inhibition of COX, and ease the hyperalgesia caused by surgical trauma (Sugimoto et al., 2016). Lipid microsphere preparation has a stronger efficacy, a faster onset, and a longer duration, and is less likely to cause gastric mucosal injury and other adverse reactions (Fukumoto et al., 2018). However, its analgesic and anti-inflammatory effects have not been confirmed through a local injection. This study investigated the safety and efficacy of Flurbiprofen Ester microspheres by a local intramuscular injection in mice, which might provide a preclinical reference for a periarticular injection of this compound.

## Materials and Methods

### Animals

The use of rats conformed to the Guiding Principles in the Care and Use of Animals approved by the Council of the China Physiology Society and was approved by the Ethics Committee of Peking Union Medical College Hospital (approval number XHDW2017-022). Three-month-old male SD rats (body weight approximately 200 g) were used and maintained under climate-controlled conditions on a 12-h light-dark cycle at 22–24°C with a relative humidity of 50%–55%.

### Drugs and Reagents

Flurbiprofen Ester microspheres were purchased from Beijing Taide pharmaceutical co., ltd. (Beijing, China). Cyclooxygenase 1 Assay Kit (HL199), Cyclooxygenase 2 Assay Kit (H200), C-reactive protein Assay Kit (H126), Interleukin -6 Assay Kit (H007) and Tumor Necrosis Factor-α Assay Kits (H052) were purchased from Nanjing Jiancheng Bioengineering Institute (China). Hematoxylin (517-28-2) and eosin (15086-4-9) were purchased from Bmresco. An invert microscope (NIKON CI-S) was purchased from NIKON (Japan).

### Surgical Technique for the Induction of a Closed Femoral Shaft Fracture

Male SD rats (12 per group) were subjected to a closed bilateral femoral shaft fracture of the right leg with femoral shaft puncture fixation followed by the laboratory anesthesia program with ketamine (40 mg/kg) (Wanfu Biological Technology Co., Ltd. Changshu, Jiangsu China) and toluene thiazide (5 mg/kg) (Fuzhou Tengyuan Biological Co., Ltd. Fujian, China), the left leg was not treated (Gilley, 2002; Freeman et al., 2008).

### Experimental Design and Flurbiprofen Ester Microspheres Injection

The 72 animals were randomly allocated into six groups. The blank control group (Group A) animals (*n* = 12) were normal rats, and an equal amount of saline solution was injected; the negative control group (Group B) animals were with a fracture (*n* = 12), and an equal amount of saline solution was injected; the positive control group (Group C) animals were with fracture (*n* = 12) and Flurbiprofen Ester microspheres (4.5 mg/kg) were injected intravenously once immediately after the fracture and 24 h after the fracture, respectively; in the three experimental groups (*n* = 12, respectively Group D, E, F), Flurbiprofen Ester microspheres (2.25 mg/kg, 4.5 mg/kg, 12 mg/kg) were injected into the adductor muscle of the fractured lower limb immediately after the fracture, and then intravenous injected (4.5 mg/kg) once 24 h later.

After the intravenous injection site was tail vein of the rat, the needle diameter was 1 ml syringe, the dilution was saline (50 mg + 50 ml) for 55 ml, the drug specifications was 5 ml/50 mg; the injection volume was 1 ml. Day 1, day 3, and day 7 after administration, four rats were taken from groups A, B, C, D, E, and F, respectively. The serum was obtained through the abdominal aorta of rats to detect IL-6, TNF-α, CRP and other indicators and evaluate the effect of systemic inflammation; the muscle tissue at the injection site was taken for pathological detection and the staining to understand the muscle necrosis.

### Preparation and Treatment of Specimens

Serum was taken after the fractures and the injection of Flurbiprofen Ester microspheres at different times. The rats were killed at different time points (24, 48, and 96 h after the operation) through an injection of 10–15 ml of air in the syringes. The muscle tissue was then immediately taken from the injection site. Finally, the muscle tissue was fixed in a 4% neutral formalin solution and prepared for the following HE stain analysis.

### Radiography Analysis

The X-ray procedures were similar to that of previous studies (Yang et al., 2015). Briefly, fracture morphology was detected by digital X-ray equipment (Siemens, Chicago, USA).

### Behavioral Assessments

After surgery at 1 h, the cumulative pain score was selected (Møiniche et al., 2002), every 5 min as a point. In order to ensure the objectivity of the score, the observer did not know the details of the experimental group. Instead of real-time observation, the observer continuously photographed the animal behavior, and then the observer scored the pain behavior of the rats through the video. It was very convenient to record the time and duration of rat action on the video tape. Finally, an objective pain score was obtained. The pain behavior was divided into four grades: the double hind feet bisect the ground, no abnormal activity-score 0; fracture side palmar slight contact with the ground, activities with claudication-score 1; fracture side foot lift, do not contact the ground-score 2; the rats licked, bit or twitched their fractured feet-score 3. Then we evaluated the behavior of the rats by pain intensity scoring (PIS), the calculation method as PIS=(T1+2*T2+3*T3)/5*60, T1, T2, and T3 respectively at the appearance time (second) of level 1, 2, and 3 at 5 min.

### Enzyme-Linked Immunosorbent Assay

1.5 ml blood of each mouse was taken to be centrifuged under the condition of 4°C, 3,000 r/min, and a 10 cm centrifugal radius for 10 min, and then the blood was stored at 20°C. The contents of inflammatory factors such as Cyclooxygenase 1 (COX-1), Cyclooxygenase 2 (COX-2), C-reactive protein (CRP), Interleukin -6 (IL-6) and Tumor Necrosis Factor-α (TNF-α) were detected by an enzyme-linked immunoassay kit (Anderson et al., 1996). All the operations were strictly carried out in accordance with the kit instructions.

### Hematoxylin-Eosin Staining

After treatment with different amounts of the drug, the muscle tissue was fixed with 4% paraformaldehyde for 48 h, then, the samples were embedded in paraffin, cut into 4 mm-thick sections and dehydrated with gradient ethanol. After that, the sections were stained with HE and assessed with the optical microscope (Zhang et al., 2019).

### Data and Statistical Analysis

All data were presented as mean (95% confidence interval). The data passed normality and equal variance tests and was analyzed by one-way ANOVA followed by an unpaired Student’s t-test with a multiple comparison post hoc test. A significant difference was defined as a *p* < 0.05.

## Results

### Closed Femoral Shaft Fracture Model

**FIGURE 1:**
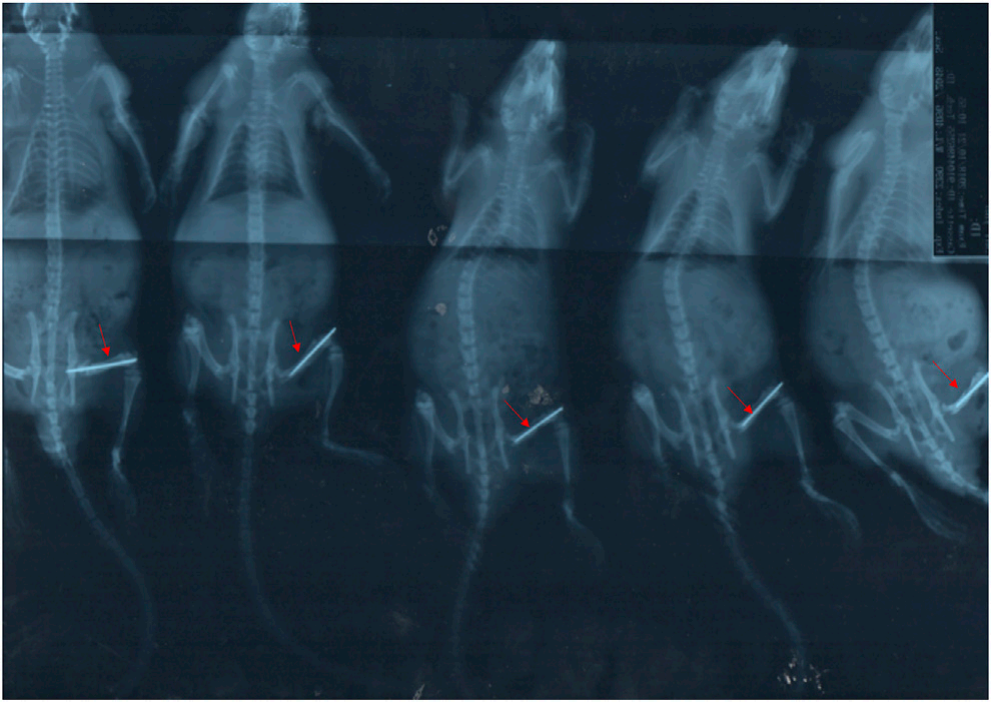
X-ray of the closed femoral shaft fracture Model.

Through X-ray testing, we could see that the fracture model was successfully established, as shown in Figure 1, and the other experiment could be followed up.

### Behavioral Test

**FIGURE 2:**
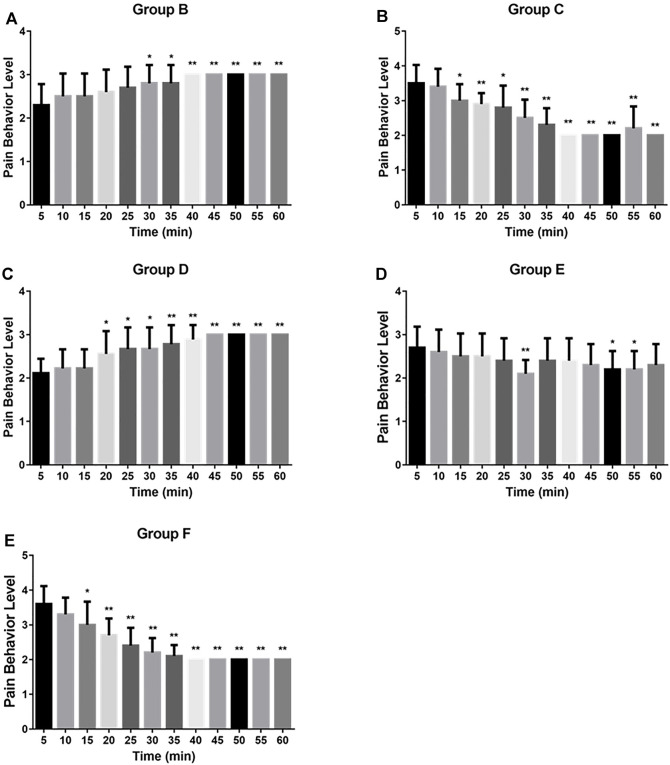
The pain behavior level of rats by different flurbiprofen ester microspheres delivery system in 60 min. **(A)** The pain behavior level of negative group in 60 min; **(B)** The pain behavior level of positive group in 60 min; **(C)** The pain behavior level of experimental group (2.25 mg/kg) in 60 min; **(D)** The pain behavior level of experimental group (4.5 mg/kg) in 60 min; **(E)** The pain behavior level of experimental group (9 mg/kg) in 60 min.

**FIGURE 3:**
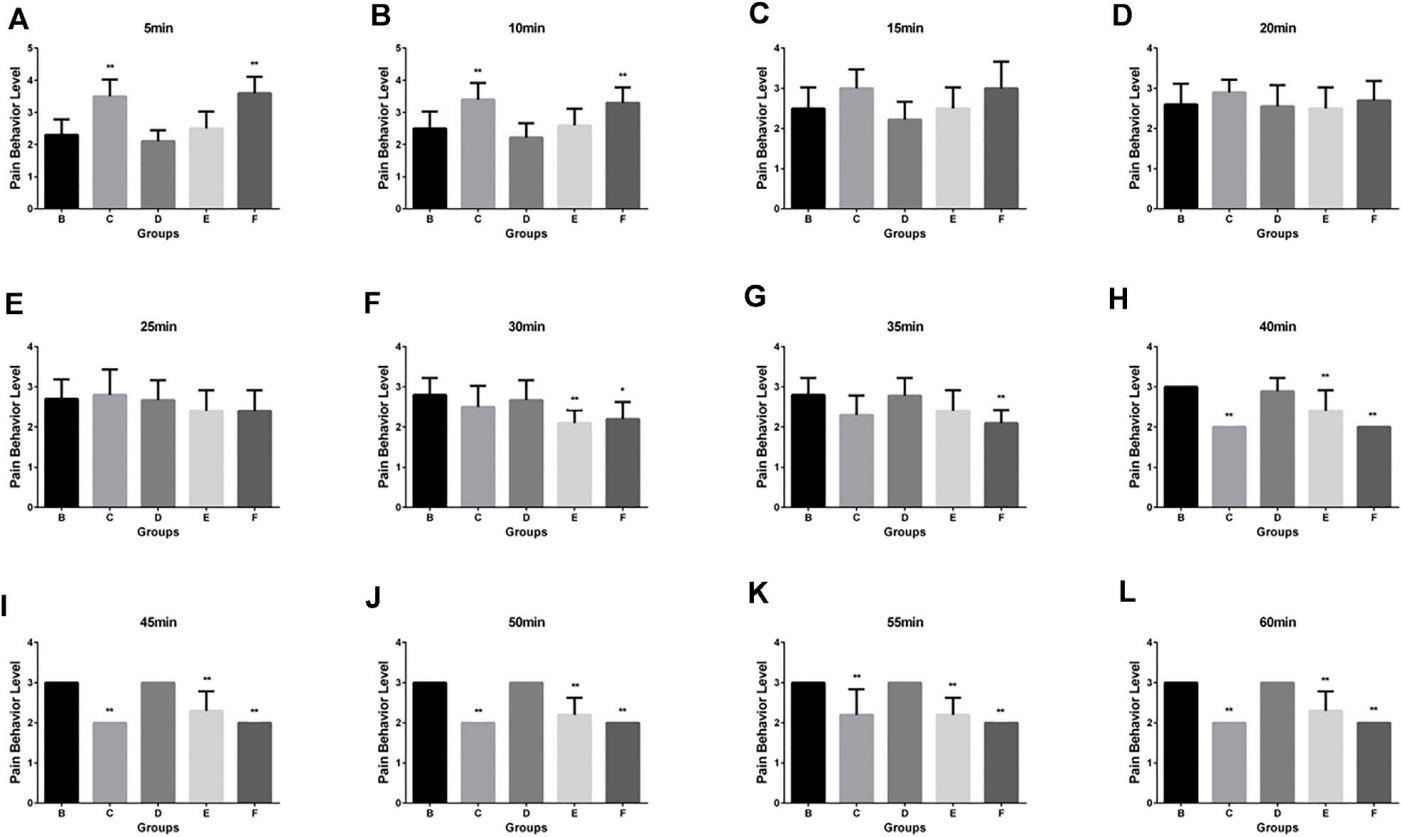
The pain behavior level by different delivery system in different time. **(A)** The pain behavior level by different delivery system in 5 min; **(B)** in 10 min; **(C)** in 15 min; **(D)** in 20 min; **(E)** in 25 min; **(F)** in 30 min; **(G)** in 35 min; **(H)** in 40 min; **(I)** in 45 min; **(J)** in 50 min; **(K)** in 55 min; **(L)** in 60 min.

The scoring results of the pain learning test are shown in the following Table 1. Through the analysis of the regular mice in the model group, we found that with the extension of modeling time, the pain of the mice significantly increased, and the pain reached the maximum intensity at 40 min, which initially indicated that our model was successfully established (Figure 2A). Then, we made the comparison in the intra group within 60min. The pain was alleviated by an intravenous injection of Flurbiprofen Ester microspheres (4.5 mg/kg); after 15 min, the pain was noticeably relieved and at 40 min, there was no change, which had a certain statistical significance (*p* < 0.05) as shown in Figure 2B. From Figure 2E, we can see that the pain could diminish with the amount of Flurbiprofen Ester microspheres (9 mg/kg) in the muscular injection, having a similar effect to the intravenous injection (*p* < 0.05), and the pain level was reduced by a muscular injection of Flurbiprofen Ester microspheres (4.5 mg/kg), but without statistical significance, excluding 30, 50, and 55 min (Figure 2D). The pain level could not be relieved by a Flurbiprofen Ester microsphere muscular injection (2.25 mg/kg) (Figure 2C). Finally, we compared the pain levels in different groups at the same time. We found that the pain level from a muscular injection or intravenous injection of Flurbiprofen Ester microspheres was not reduced at 20 min, and the pain even became more serious than the negative control group (Figures 3A–D). From 25 min, the pain began to subside (Figures 3E–L), however, the pain was not reduced by a muscular injection of Flurbiprofen Ester microspheres (2.25 mg/kg) compared to the negative control group (Figures 3E–L). Compared to the negative control group, the pain was reduced significantly by a muscular injection of Flurbiprofen Ester microspheres (4.5 mg/kg and 9 mg/kg) from 30 min (Figures 3F–L), and through the intravenous injection of Flurbiprofen Ester microspheres (4.5 mg/kg), from 40 min (Figures 3H–L). There wasa similar effect when amounts of Flurbiprofen Ester microspheres of 4.5 mg/kg and 9 mg/kg were injected intravenously and into the local muscular respectively, but there was no significant difference. So, as concluded in the behavioral test, we noticed that the effect of the intramuscular and intravenous injections of Flurbiprofen ester microspheres was similar.

**TABLE 1:** The score of behavioral test.

Time (min)	Group B		Group C		Group D		Group E		Group F	
	Ave	SD	Ave	SD	Ave	SD	Ave	SD	Ave	SD
5	2.3	0.5	3.5	0.5	2.1	0.3	2.7	0.5	3.6	0.5
10	2.5	0.5	3.4	0.5	2.2	0.4	2.6	0.5	3.3	0.5
15	2.5	0.5	3.0	0.5	2.2	0.4	2.5	0.5	3.0	0.7
20	2.6	0.5	2.9	0.3	2.6	0.5	2.5	0.5	2.7	0.5
25	2.7	0.5	2.8	0.6	2.7	0.5	2.4	0.5	2.4	0.5
30	2.8	0.4	2.5	0.5	2.7	0.5	2.1	0.3	2.2	0.4
35	2.8	0.4	2.3	0.5	2.8	0.4	2.4	0.5	2.1	0.3
40	3.0	0.0	2.0	0.0	2.9	0.3	2.4	0.5	2.0	0.0
45	3.0	0.0	2.0	0.0	3.0	0.0	2.3	0.5	2.0	0.0
50	3.0	0.0	2.0	0.0	3.0	0.0	2.2	0.4	2.0	0.0
55	3.0	0.0	2.2	0.6	3.0	0.0	2.2	0.4	2.0	0.0
60	3.0	0.0	2.0	0.0	3.0	0.0	2.3	0.5	2.0	0.0

### The Expression of Inflammatory Cytokines by ELISA

**FIGURE 4:**
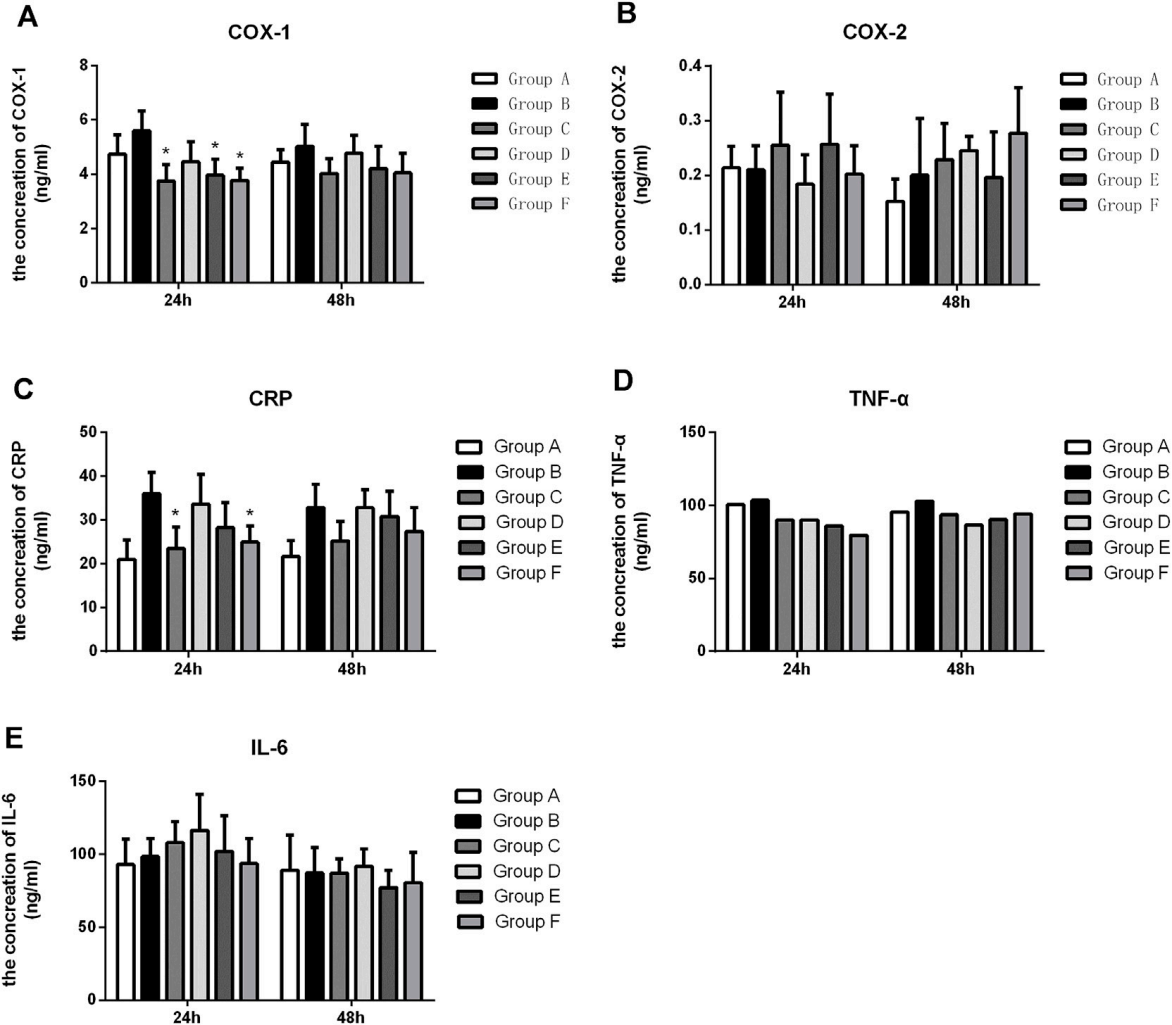
The level of COX-1, COX-2, CRP, TNF-α and IL-6 in the serum after treatment with flurbiprofen ester microspheres by different delivery system. **(A)** The level of COX-1 in the serum by different treatment of flurbiprofen ester microspheres; **(B)** the level of COX-2 in the serum by different treatment of flurbiprofen ester microspheres; **(C)** The level of CRP in the serum by different treatment of flurbiprofen ester microspheres; **(D)** The level of TNF-α in the serum by different treatment of flurbiprofen ester microspheres; **(E)** The level of IL-6 in the serum by different treatment of Flurbiprofen ester microspheres, *p < 0.05.

We further investigated whether Flurbiprofen Ester microspheres affected the expression level of inflammatory cytokines when the different delivery systems were used. Our results showed that the level of COX-1 in groups C, D, E, and F was significantly lower compared to the negative control group at 48 h, but only groups C, E and F had a significant difference with group B (*p* < 0.05) at 24 h, which indicated an intravenous and high-dose local administration of Flurbiprofen Ester microspheres can inhibit the production of COX-1. When comparing this to the intravenous (Group C), the high dose of Flurbiprofen Ester microspheres through intramuscular injection (Group F) had no significant difference at each time point. These results showed that local drug delivery was effective (Figure 4A). However, for COX-2, we did not achieve a positive result (Figure 4B). Then, when we tested the expression of CRP, we found that the serum level of CRP at 48 h was increased in the rats with femoral shaft fractures. When treated with Flurbiprofen Ester microspheres, the serum level of CRP decreased at 48 h. However, in the rats treated with an intravenous injection of Flurbiprofen Ester microspheres (4.5 mg/kg) and a muscular injection of Flurbiprofen Ester microspheres (9 mg/kg), the serum level of CRP was significantly reduced (*p* < 0.05) at 24 h. This demonstrated the effectiveness of the intramuscular injection (Figure 4C). Also, the serum levels of TNF-α were reduced in groups C, D, E and F compared to group B, but without a significant statistical difference (Figure 4D). Finally, we found that the level of serum IL-6 increased after fracture at 48 h (Figure 4E). These findings suggested that a local muscle injection of Flurbiprofen Ester microspheres had a similar effect to that of an intravenous injection.

### Histochemistry of the Muscle Tissue for the Rats

**FIGURE 5:**
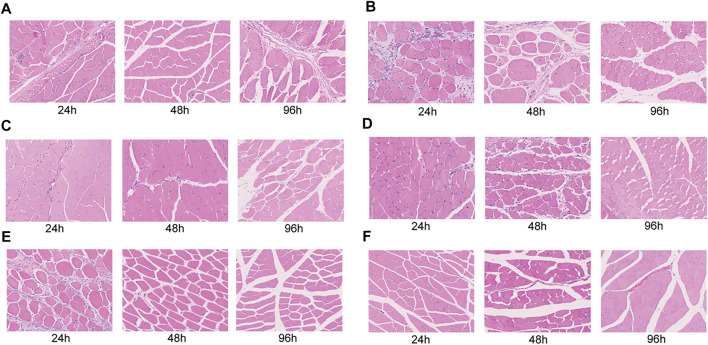
Histopathological evaluation of the leg muscular tissue after injected flurbiprofen ester microspheres by intramuscular and intravenous at 24, 48, and 96 h. **(A)** The blank control of normal rats injected saline solution; **(B)** The negative control of the closed femoral shaft fracture injected saline solution; **(C)** The positive control of the closed femoral shaft fracture by intravenous injection flurbiprofen ester microspheres 4.5 mg/kg; **(D)** The experiment group of the closed femoral shaft fracture by intramuscular injection flurbiprofen ester microspheres 2.25 mg/kg and; **(E)** 4.5 mg/kg; **(F)** 9 mg/kg.

The histochemistry (HE) analysis (24, 48, 96 h) of the muscle tissue showed no muscle necrosis in the normal group, but obvious necrosis in the closed femoral shaft fracture model group (Figures 5A,B). After treatment of Flurbiprofen Ester microspheres by intramuscular injection at different doses, there was no obvious muscle necrosis (Figures 5C–F).

## Discussion

After intravenous administration, Flurbiprofen Ester microsphereare rapidly hydrolyzed by enzymes in the blood into the active metabolite Flurbiprofen, which can obtain a high blood concentration and produce a rapid and powerful analgesic effect (Sun et al., 2013; Lin et al., 2014). Like other NSAIDs, Flurbiprofen blocks the biosynthesis of prostaglandins by inhibiting COX activity, thus playing an analgesic role (Zhou et al., 2019). The dispersion system of nano-level particles that form when drugs are dissolved or dispersed in the matrix of lipid components is called the lipid microsphere (Sznitowska et al., 2017). The lipid microsphere is an ideal drug carrier due to its excellent safety, stability and biocompatibility. It can significantly reduce drug irritation and adverse reactions (Jaspart et al., 2005; Wosicka-Frąckowiak et al., 2015). The drug is encapsulated in the particle and can be released continuously. Also, the drug is good at targeting, has a particle size of about 200 nm, and will not block the capillary (Weber et al., 2014). Moreover, it can accumulate in tissues with leaky vessels (such as tumors and inflammation), which means an enhanced permeability and retention (EPR) effect (Kobayashi et al., 2013). Thus, the lipid microspheres had a natural passive targeting effect. Due to the targeted impact of Flurbiprofen Ester microspheres, we hypothesized that a local injection of Flurbiprofen Ester microspheres could rapidly act around the wound with the most severe inflammatory response, and more effectively reduce the local inflammatory response to alleviate pain, and to reduce the systemic inflammatory response through systemic absorption. However, the safety of a local injection has not been reported on in previous studies.

The lower limb anatomical structure of SD rats is clear, and the modeling process is relatively simple, with excellent model stability. At present, the femoral shaft closed fracture modeling method is a ripe model (Xing et al., 2013) that is simple to operate and easy to create and replicate, and the systemic inflammation model is closer to the clinical disease model (Weckbach et al., 2012). So, in this study, the safety and efficacy of a local injection of Flurbiprofen Ester microspheres was evaluated in this model.

The pain behavioral test is a required method to assess the safety and efficacy of the drug. Our results showed that an intramuscular injection of Flurbiprofen Ester microspheres was effective compared to the negative control group, while compared to the positive control, there was no obvious difference. Hence, intramuscular and intravenous injections of Flurbiprofen Ester microspheres had a similar analgesic effect.

To further evaluate the effect of an intramuscular injection of Flurbiprofen Ester microspheres, we detected the systemic inflammatory factors COX-1, COX-2, CRP, TNF-α and IL-6. NSAIDs can interfere with the metabolism of arachidonic acid by inhibiting the activity of COX, reduce the production of prostacyclin synthase, and then decrease the synthesis of prostaglandin, relieving the pain sensation after surgery, and thus performing an analgesic role (Seibert and Masferrer, 1994; Sinatra, 2002). Our result showed that the levels of COX-1 in groups C, D, E, and F were significantly lower compared to the negative control group at 48 h. Still, only groups C, E and F showed a significant difference (*p* < 0.05) at 24 h, which indicated the intravenous and high-dose local administration of Flurbiprofen Ester microspheres could inhibit the production of COX-1. But these two groups had no significant difference at every time point. These results confirmed the effectiveness of local drug delivery (Figure 4A). However, for COX-2, we did not achieve a positive result (Figure 4B). It may be that Flurbiprofen Ester microspheres mainly acted on COX-1 but had little effect on COX-2. In addition, the increase of COX-2 indicated that there were still other factors that affected its production. Thus, the specific mechanism needs further study.

CRP is an acute-phase protein, which is a plasma protein produced by the liver that rises sharply during infection or tissue damage (Kawai, 2000). As an acute protein, CRP is induced in synthesis by inflammatory mediators, and its elevation is closely related to the degree of inflammatory injury (Rückerl et al., 2007). Our research found that the serum level of CRP at 24 h was increased in the rats with femoral shaft fractures. This indicated that CRP as markers of an inflammatory response steadily increased after surgery, and it was sensitive to the fracture strike, which can reflect the inflammatory response state of the body to some extent. Rats in groups C and F were treated with Flurbiprofen Ester microspheres, and compared to group B, CRP showed a significant decrease with a statistical difference (*p* < 0.05) at 24 h. The result proved that Flurbiprofen Ester microspheres can effectively reduce systemic the inflammatory response in both intravenous and topical use. In groups D, E and F, compared to group C, there was no significant difference, but group F showed a similar effect to group C, which demonstrated the effectiveness of the intramuscular injection (Figure 4C). The abnormal increase of CRP illustrated that there were other factors that influence its production, and that the mechanism needs further research.

TNF-α is produced by mononuclear macrophages and is involved in fever, inflammation and immune regulation (Fukaya et al., 2013). TNF- plays an important role in the physiological function, immune regulation and anti-infection process of the body (Bauer et al., 1996). We discovered that the serum level of TNF-α in groups C, D, E and F was without significant statistical difference compared to group B (Figure 4D). However, the level of TNF-α decreased in all treated groups, which indicated that Flurbiprofen Ester microspheres played a role in fractures when injected intravenously and intramuscularly.

Il-6 is a potent inflammatory cytokine and is at present generally regarded as one of the indicators reflecting the inflammatory state of the body (Hoffmann et al., 1994). Our result found that the level of serum IL-6 increased after fracture at 24 h, the level of IL-6 was reduced only through an intramuscular injection of Flurbiprofen ester microspheres (9 mg/kg), however, the level of IL-6 increased with an intravenous (4.5 mg/kg) and intramuscular injection (2.25 mg/kg and 4.5 mg/kg), which showed IL-6 was more sensitive to a fracture and can reflect the body’s inflammatory response to a certain extent (Figure 4E).

We found that an intramuscular injection of Flurbiprofen Ester microspheres of 2.25 mg/kg, 4.5 mg/kg and 9 mg/kg did not cause muscle necrosis compared to the negative and positive control group in histochemistry (Figure 5). This result indicated that Flurbiprofen Ester microspheres are safe through local drug delivery.

This study tried to simulate the clinical practice of a local injection of Flurbiprofen Ester microspheres and tried to verify the effectiveness of Flurbiprofen Ester microspheres by detecting the pain behavior of the model rats and the inflammatory indicators in the serum of rats. Due to the individual differences of rats, the detected indicators were biased, and statistical analysis could not be conducted completely. However, the design of this study was rigorous. The sample size of each group was 12, and the negative control group and the blank control group can ensure sufficient statistical efficiency to explain the problem. On the other hand, the behavioral test (after modeling) can better illustrate the effectiveness of the use of Flurbiprofen Ester microspheres. Studies also showed that the detection of inflammatory factors and COX can be used to illustrate the effectiveness, and the results were objective and reliable. How to combine the two ways to illustrate the effectiveness of the local application of Flurbiprofen Ester microspheres can be further studied.

There are still some limitation in this study. Firstly, Lack of research on the influence of formula particle size, and lack of particle characterization of formula, such as SEM or TEM, XRD and incompatibility. In addition, Due to limited research funding, we may not be able to complete morphological experiments such as scanning electron microscopy for this drug.

In conclusion, Flurbiprofen Ester is a new type of NSAIDs, its local injection significantly reduced the inflammatory response in rats with systemic inflammation of closed femoral shaft fractures. Also, it did not increase the risk of muscle necrosis, suggesting the feasibility of its application in local injection analgesia.

## Data Availability

The original contributions presented in the study are included in the article/supplementary material, further inquiries can be directed to the corresponding author.
